# Polyurethane Foams Loaded with Carbon Nanofibers for Oil Spill Recovery: Mechanical Properties under Fatigue Conditions and Selective Absorption in Oil/Water Mixtures

**DOI:** 10.3390/nano11030735

**Published:** 2021-03-15

**Authors:** Annamaria Visco, Antonino Quattrocchi, Davide Nocita, Roberto Montanini, Alessandro Pistone

**Affiliations:** 1 Department of Engineering, University of Messina, C.da di Dio (S. Agata), I-98166 Messina, Italy; antonino.quattrocchi@unime.it (A.Q.); roberto.montanini@unime.it (R.M.); 2 Institute for Polymers, Composites and Biomaterials-CNR IPCB, Via P. Gaifami 18, I-95126 Catania, Italy; 3 Polymer IRC, Faculty of Engineering and Informatics, University of Bradford, Bradford BD7 1DP, UK; D.Nocita@bradford.ac.uk

**Keywords:** polyurethane foams, carbon nanofiller, oil spill recovery, selective adsorption, fatigue

## Abstract

Marine pollution due to spillage of hydrocarbons represents a well-known current environmental problem. In order to recover the otherwise wasted oils and to prevent pollution damage, polyurethane foams are considered suitable materials for their ability to separate oils from sea-water and for their reusability. In this work we studied polyurethane foams filled with carbon nanofibers, in varying amounts, aimed at enhancing the selectivity of the material towards the oils and at improving the mechanical durability of the foam. Polyurethane-based foams were experimentally characterized by morphological, surface, and mechanical analyses (optical microscopy observation, contact angle measurement, absorption test according to ASTM F726-99 standard and compression fatigue tests according to ISO 24999 standard). Results indicated an increase in hydrophobic behavior and a good oleophilic character of the composite sponges besides an improved selective absorption of the foam toward oils in mixed water/oil media. The optimal filler amount was found to be around 1 wt% for the homogeneous distribution inside the polymeric foam. Finally, the fatigue test results showed an improvement of the mechanical properties of the foam with the growing carbon filler amount.

## 1. Introduction

In recent years a problem which has attracted more and more attention has been hydrocarbon spillage into sea-water. During exploitation or transportation, oil spills occur frequently, causing serious damage to marine organisms, thus becoming a major global environmental problem. Common methods employed to recover spilled oils include physical adsorption [1], chemical treatment in situ [2], combustion [3], and biotechnologies [4]. The aim of any recovery operation is to collect as much oil as is reasonably and economically possible. A successful recovery system must overcome the interrelated problems of encountering significant quantities of oil and its subsequent containment, concentration, recovery, pumping and storage. From this point of view, physical adsorption meets these requirements because it represents an easy and cheap method. It is simple to operate with automated skimmers vessels, allowing to pump the oil to storage.

Physical adsorption needs a suitable adsorbent material. Many natural sorbents, like sawdust [5], wool fiber [6], and zeolite [7], have been investigated, but the main limitations are represented by the low oil selectivity for oil/water systems and very limited recyclability. A porous polymeric composite is considered as an effective adsorption material because it is easy to prepare and to recycle, it has a satisfactory adsorption capacity and a good oil selectivity after opportune tuning of hydrophobic and hydrophilic chemical structure of the surface [8,9,10,11]. To date, several polymer grades have been employed as oil adsorbents, such as foams [12,13], resins [14,15,16,17], sponges [18], and aerogels [19,20]. Amongst the different polymer matrices, polyurethane foams have recently attracted great attention for oil/water separation processes [21,22]. However, the polar groups such as carboxyl, as well as amino groups, present on polyurethane frameworks, make these materials hydrophilic, thus reducing their selective nature and their overall performance [22].

Consequently, recent studies were focused on the surface modification of the polyurethane sponges: the goal was to achieve hydrophobic surfaces so that the oil absorption capacity during the oil/water separation process would increase. Several materials were used to switch the hydrophilic sponge surface to hydrophobic, such as Fe_3_O_4_ [23], SiO_2_ [24], ZnO [25], Ag [26], making commercial sponges promising candidates for crude oil removal both from seawater and industrial wastewater. Amongst carbon-based materials used for the hydrophobic functionalization of polyurethane sponges, carbon nanoparticles, graphene or carbon nanotubes have been employed as fillers for polyurethane-based sponges.

Shi et al. [27] fabricated carbonaceous nanoparticles (CNPs) modified polyurethane (PU) foam using the ultrasonication technique. The CNPs-PU foam showed absorption of organic solvents up to 50–121 times its own weight and could withstand 500 cycles of compression at 80% compressing strain without any plastic deformation. Polyurethane sponges coated with graphene or reduced graphene oxide were also investigated, reaching absorption capacity up to 39 and 80–160 times its own weight, respectively after 120 or 50 cycles [28,29]. Wang et al. [30] synthesized carbon nanotubes (CNTs) reinforced polyurethane (PU) sponge whilst measuring absorption capacity up to 35 times of its own weight after 150 cycles. Keshavarz et al. [31] developed a surface modification method enhancing the light crude oil sorption capacity of polyurethane foam (PUF) through immobilization of multi-walled carbon nanotube (MWCNT) on the foam surface. They achieved a 21% enhancement in light crude oil sorption compared to the blank PUF.

Within the carbon nanomaterials family, carbon nanofibers (CNF) were not widely explored for oil/water separation applications with respect to graphene or carbon nanotubes [32,33]. Carbon nanofibers have displayed excellent hydrophobic characteristics coupled with very cheap synthesis costs if compared to other carbon-based nanomaterials. Zhen-Yu Wu et al. [34] synthesized carbon nanofiber aerogels by means of a freeze-drying method with multiple steps; the prepared pure CNF aerogel demonstrated good oil sorption ability. Yang et al. [35] fabricated an MCF (multi-functional carbon fiber) aerogel by using natural bamboo chopsticks as raw material. A porous structure was obtained amongst the neighboring fibers, with the diameter of fibers ranging from 8 to 10 nm. The MCF aerogel showed a moderate oil uptake capacity (~80 g/g) in comparison with some ultra-flyweight synthetic aerogels. Nevertheless, the material can be recycled easily using distillation, combustion, or squeezing, due to its porous and hydrophobic nature. Moreover, it showed high thermal and mechanical stability.

The combination of CNF with porous supports can make it a valuable material for the oil/water separation. Polyurethane sponge is of porous nature and provides a good opportunity liquids absorption. Its wettability can be improved by modifying its porous network with low surface energy hydrophobic materials [36]. The combination of CNF and polyurethane foam can be successfully employed to produce hydrophobic sponges for cleaning and absorbing the oil from contaminated waters. Baig and coworkers [37], recently utilized the dip coating technique to load PU sponge with carbon nanofibers (CNF). The porous network of polyurethane provided enough space for the selective absorption of oil from water, exhibiting large surface area, and small pore size. The process of grafting CNF onto polyurethane enhanced the hydrophobicity of the sponge and increased the number of pores which might be responsible for capillary action, efficiently removing oil and other organic contaminants from water. The synthesized CNF grafted PU showed excellent performance in separating non-polar organic contaminants and oil from water in both static and dynamic conditions showing a good mechanical stability. 

In this paper we studied polyurethane sponges grafted with hydrophobic carbon nanofibers, synthesized for the selective recovery of oil from water. The analyzed material offers the advantages of an easy production process and of the low raw materials cost. Furthermore, particular attention was given to the study of the fatigue behavior of the polyurethane-CNF sponges. Fatigue properties of polymers and composites still represent a timely topic, which has not been widely investigated [38]. However, in many fields of application, the response of these materials to dynamic forces allows one to obtain useful information [39,40]. In the case of polyurethane materials, for example, Wei et al. [41] investigated the performance of rigid polyurethane grout, applied for the maintenance of infrastructure, identifying the failure mode under compression load. Calvet et al. [42] studied commercial grades of rigid polyurethane foams, commonly used to mimic trabecular bone in testing orthopedic devices. They observed how the hysteresis can be used as an additional parameter structural design. Baig et al. [37] tested the absorption of their prepared material using a dynamic system. Mechanical fatigue properties of polyurethane foams have been well known for more than 30 years [43,44]. However, in the field of oil recovery, the literature is lacking on dynamic evaluations [45].

## 2. Materials and Methods

### 2.1. Materials

The reagents used for polyurethane foam synthesis were parts of a bicomponent (A and B) ESPAK SOFT PU foam kit (Prochima srl, Pesaro-Urbino, Italy). The main constituents of the two components were: Methylene diphenyl diisocyanate oligomers (MDI) and polyether polyols (PEP) as polymerizing cross-linking elements; tertiary aliphatic amines as catalysts; water as blowing agent and silicone-based surfactants. This commercial product is used to produce foams with density of ca. 0.5 g/cm^3^. Chopped carbon nanofibers were purchased by ZOLTEK (Bridgeton, MO, USA). Cyclohexane (purity ≥ 99%) and Na_2_SO_4_ (purity ≥ 99%), used for the selective absorption tests, were purchased from Sigma Aldrich (Merck KGaA, Darmstadt, Germany). Commercial diesel fuel for automotive motion (density 0.82 g/cm^3^ at 25 °C) was used to simulate spilled oil.

### 2.2. Synthesis of PU Foams-Based Composites

**Figure 1:**
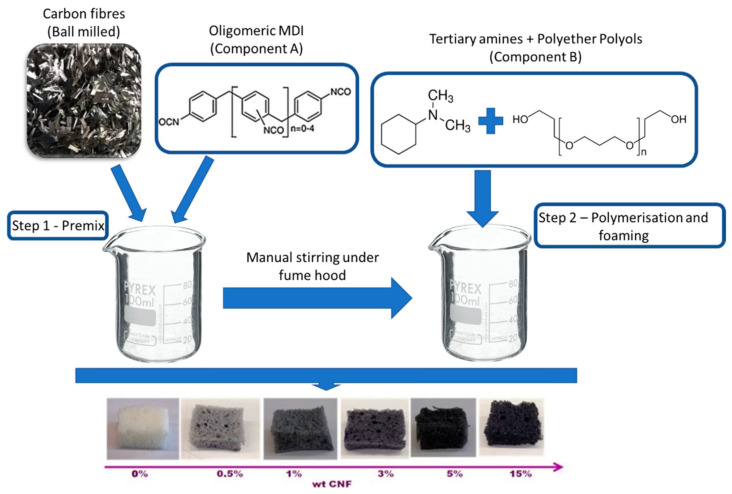
Synthetic route of polyurethane foams loaded with carbon fibers.

**Figure 2:**
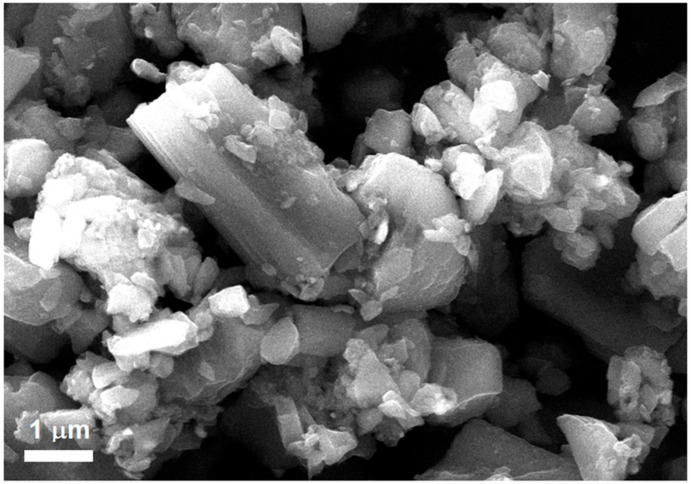
SEM analyses of carbon fibers after the ball milling treatment. Dimension 1 µm, magnification 87,000×.

Polyurethane foams were synthesized by mixing the components A and B in a 2:1 ratio, according to the supplier directions and to the scheme reported in Figure 1. In details, we mixed 10 g and 5 g of component A and B, respectively. The carbonaceous filler was obtained by crushing chopped carbon nanofibers in a high-energy ball milling (Fritsch Mini-Mill II, Idar-Oberstein, Germany) apparatus, obtaining carbon fibers with length or width < 1 µm (Figure 2). It has been employed for 5 min at a speed of 250 rpm. This dispersion cycle was repeated 25 times for each sample. The ball milling is composed by two jars whose volume is 25 mL. Spherical stainless-steel ball (inserted in each jar) has a diameter of 15 mm and a volume of 14.1 mL. Usually, 1 g of material was inserted in each jar for its mechanical treatment.

The filler was added to component A (MDI oligomers) and mixed vigorously. Subsequently, component B is poured in the mixing container, and, after 30 s the mixture is left free to foam. Polyurethane-based foams, pristine and with increasing loading of carbon nanofibers, corresponding to 0.5, 1, 3, 5, and 15 wt% (calculated on the total amount of 15 g of Component A + B) are indicated as: PUCNF0, PUCNF0.5, PUCNF1, PUCNF3, PUCNF5, and PUCNF15, respectively (see Figure 1).

### 2.3. Characterization of PU Foam-Based Composites

The carbon fibers morphology has been observed by scanning electron microscopy with a FEI Quanta mod. FEG450 (FELMI-ZFE instruments, Graz, Austria). CNF powder adhered on an aluminum holder by means of a graphitic adhesive. SEM microscope operated with an accelerating voltage of 15 kV and in low vacuum mode. The image magnification was of 87,000×.

The foams morphology was examined by Optical Microscopy (OM): an Hirox digital microscope KH 8700 was used to observe foams surface and to measure their pore maximum, minimum and average diameter. The magnification was 100×.

The foams wet-ability was measured by depositing a micro-drop of distilled water or oil (1 μL) at room temperature, on the horizontal surface of the polyurethane foams using the sessile-drop technique and measuring the Wenzel contact angle [46].

A microlithic syringe (Hamilton Company, Bonaduz (GR), Switzerland, 10 μL), was used to deposit the fluid drop and a video camera connected to the computer captured the images of drops as soon as they were deposited on the material. A software (GIMP Image Manipulation Program) allowed the measurement of the baseline of each drop. The following Equation (1) was then used to evaluate the contact angle:

*θ_w_* = 2 *arctg* (2 *h*/*d*), (1)

where *θ_w_* is the Wenzel’s contact angle of the surface, *d* and *h* are the two geometrical parameters of each fluid drop discussed above. Each value is the resulting of ten measurements calculated with their standard deviations.

The water and oil absorption capacities (*C_water_* and *C_oil_*) were obtained after immersing the foams in pure water or pure diesel fuel. Oil and water absorption capacities of pristine and carbon nanofiber loaded foams were determined with samples of 1 cm^3^ in contact with 50 mL of the considered liquid for 30 min, under stirring, according to the ASTM F 726-99 standard; samples were left to drip for 30 s before weighing. All these values are expressed as the mass of the absorbed liquid divided by the mass of the absorbent (g/g). *C_water_* and *C_oil_* were calculated using Equations (2) and (3), respectively:

*C_water_* [g/g] = (*w* (*wet foam*) − *w* (*dry foam*))/*w* (*dry foam*), (2)

*C_oil_* [g/g] = (*w* (*wet foam*) − *w* (*dry foam*))/*w* (*dry foam*), (3)

where *w (dry foam)* is the initial weight of the foams, and *w (wet foam)* are the weights of the foams after the absorption of water or diesel fuel, respectively.

The selectivity towards diesel fuel absorption was also measured by immersing the foam samples (1 cm^3^) in a water/diesel mixture containing 10 vol% of diesel fuel for 30 min, under stirring. Then samples were left to drip for 30 s and the amount of liquid mixture l absorbed was obtained by weighing. Samples were subsequently washed with cyclohexane to extract the oil absorbed. The washing liquid was treated with Na_2_SO_4_ to remove the traces of water present. The final mixture was then filtered, and cyclohexane was removed by rotavapor. These steps allowed to determine the diesel fuel amount absorbed by the foams in the water/diesel oil emulsion. The amount of water absorbed in the water/diesel fuel solution was instead obtained by difference with the weight of the foams immediately after the first step (absorption of water/oil mixture). The water and oil absorption capacities in the emulsion (*C_water,emul_* and *C_oil,emul_*) were calculated as in Equations (4) and (5):

*C_water,emul_* [g/g] = (*W_fab_* − *W_or_*)/*W_f_*, (4)

*C_oil,emul_* [g/g] = *W_or_*/*W_f_*, (5)

where *W_fab_* is the weight of foams after absorption test, *W_or_* is the weight of oil after rotavapor treatment, *W_f_* is the weight of dry foam.

Fatigue tests were performed using an electromechanical testing machine (Electropuls E300, Instron, Norwood, MA, USA), equipped with two compression plates and a calibrated load cell of ±250 N. The experimental procedures were carried out according to the ISO 24999 standard. Specifically, a sinusoidal and uniaxial compression load was applied to the samples, using a frequency of 1 Hz at a temperature of 23 ± 1 °C and at a humidity of 50 ± 10 %RH for 80,000 cycles. All samples were shaped as rectangular parallelepipeds with sizes of 2.5 cm × 2.5 cm × 1 cm. Each compression step was controlled in displacement, reaching the 50% of the initial height (i.e., 0.5 cm) of the single specimen. The sampling frequency was set to 20 Hz, considering a dedicated cycle reduction mode: from 0 to 1000th cycle all cycles were captured, while between 1000th and 80,000th only one cycle in every four was recorded.

## 3. Results and Discussion

### 3.1. Morphologcal Analyses

**Figure 3:**
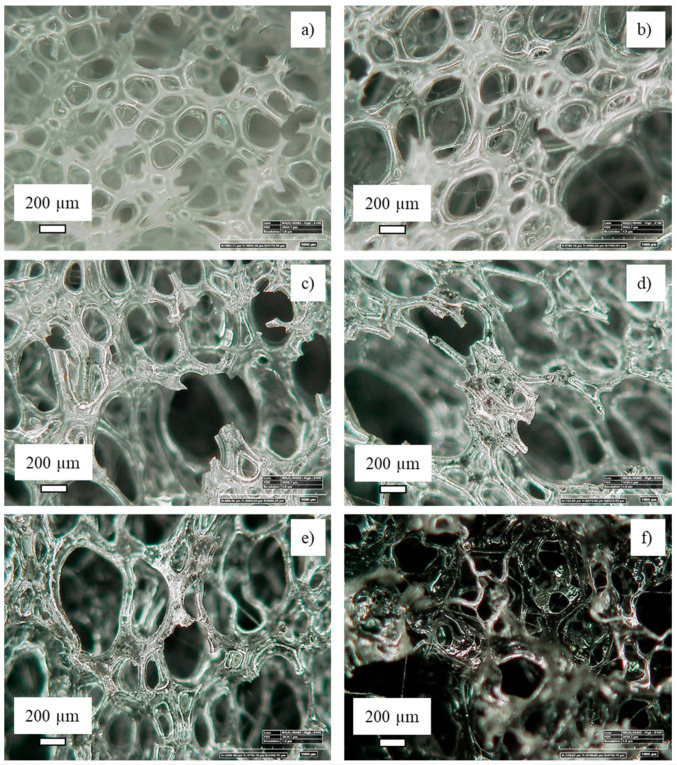
OM images of PUCNF0 (**a**), PUCNF0.5 (**b**), PUCNF1 (**c**), PUCNF3 (**d**), PUCNF5 (**e**), and PUCNF15 (**f**).

The sponge’s morphology was observed by optical microscopy (Figure 3) at 100× magnification. The pristine polyurethane sponge (sample PUCNF0) showed a uniform structure with cells of well-defined circular shape. The diameters of the cells were ca. 200 µm, some of which exhibited a “drool” due to the breaking of the bubbles during the foaming phase. The structure was transparent or slightly white.

In the composite with 0.5% by weight of carbonaceous charge (PUCNF0.5) exhibited some foam smudging. Higher magnification images showed the presence of the filler in the shape of dots throughout the structure, unevenly distributed due to the low filler content. An increase in the number of macropores can be noted for composites with 1% by weight of filler (PUCNF1) and higher. Despite the higher concentration no coalescence phenomena, leading to poor dispersion, were observed. In the PUCNF3 sample, large and elongated pores are observed while the carbonaceous particles showed some agglomeration. The PUCNF5 sample showed a substantial change in structure. The pores are elongated and deformed, losing the typical rounded shape that characterized the PU sponge or the samples PUCNF0.5 and PUCNF1. By adding a higher filler load, i.e., 15% by weight, (PUCNF15) the pores become smaller and irregularly shaped due to the excessive filler concentration. The coalescence phenomenon is accentuated, and the sample turns black.

The average size of the pores (listed in Table 1) increases by increasing the concentration of filler from 115 μm (in PUCNF0) to 190 μm (in PUCNF5), respectively. This is due to the increase in the number of bigger pores. Then the average size shrank (value of 90 μm) for the PUCNF15 foam; this is due to supersaturation conditions of the carbonaceous filler inside the polymeric matrix that hinders the natural reticulation of the polyurethane matrix.

### 3.2. Wet-Ability Measurements

The contact angle of droplets of water and oil deposited on the foams were calculated for the pristine polyurethane sponge and for the samples loaded up to 5 wt%; sample loaded with 15 wt% of filler was not considered due to the collapsed morphology highlighted by the optical microscopy images.

**Figure 4:**
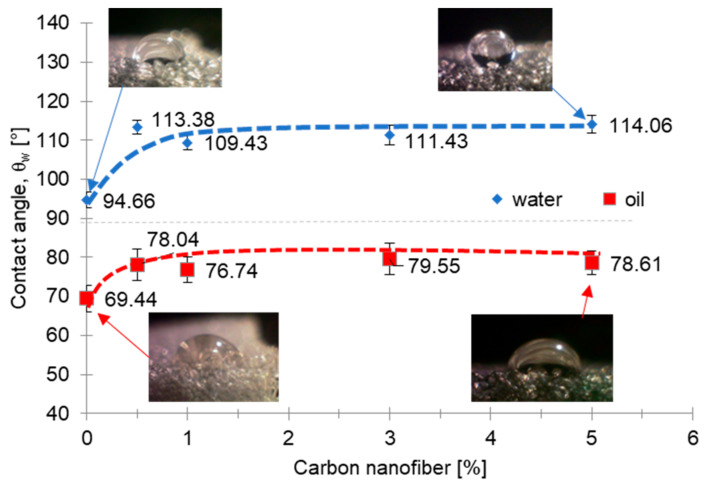
Wenzel contact angle of water and oil of the sponges at different CNF content with the image of the liquid droplets at the lowest (0%) and at the highest (5%) filler amount. Each value is the average of ten measurements and the amplitude of its error bar is two times the corresponding standard deviation.

The wet-ability tests showed how the contact angle with water and with lubricating oil increases greatly upon the addition of carbon filler in the polyurethane matrix and stayed almost constant by varying the amount of filler. The measured contact angles were always greater than 90° in the water test due to the hydrophobic behavior of the samples while, in the oil test, the contact angles were always less than 90° indicating an oleophilic surface (Figure 4).

The contact angle observed in the nanocomposite sponges indicated a low surface energy between liquid and solid as it was greater than 90°. According to the Cassie–Baxter model, this could also be due to air bubbles trapped inside the pores that do not allow the liquid to penetrate inside [47].

### 3.3. Absorption Tests

As reported in literature by Li et al. [48], the absorption test was performed according to the ASTM F 726-99 standard. For each sample, the absorption capacity of water and of oil was determined; absorption tests were also performed in a mixture of water and oil (10 v%) to quantify the absorption capacity of the samples under competitive conditions of absorption. The oil absorption properties of the samples are reported as a ratio between oil and water capacity, both in the pure systems and mixed system (Table 2).

The sample with 1 wt% of carbonaceous filler showed the lowest absorption of distilled water and the maximum absorption of diesel fuel, showing the highest Os/Ws ratio. Furthermore, PUCNF1 sample showed the highest absorption ratio between oil and water in the oil/water mixture, indicating improved selectivity for oil absorption if compared to the other samples. As listed in Table 2, the presence of CNF showed a +105% enhancement of the oil absorption capacity for PUCNF1 sample, in pure system (from 2.56 to 5.25) and of +22.85% in mixed system (from 1.75 to 2.15). Li et al. [48] studied PU samples modified by a coating with oleophilic monomer, Lauryl methacrylate (LMA) microspheres. They observed an improvement of +26.8% in diesel fuel absorption, and of +18.47% in kerosene absorption, with respect to pristine PU. Thus, our result seems to be close that of these Authors in terms of absorption capacity in mixed systems, compared to that of pure PU. These authors, referred to the ASTM F729-99 standard for the absorption capacity measurement too.

On the other hand, Baig et al. [37] found a significant enhancement in the absorption capacity of CNF grafted PU with values of 26 (g/g) for hexane, and 49 (g/g) for toluene. The same authors presented a table in which they compared their results with those of the absorbing materials of other authors (based on composite resin, foams, aerogels, etc.). They highlighted how their PU-based sponges were more efficient due to the capillary absorbing effect of the material’s particularly small pores (about 36 Å wide). The absorption values of the sponges analyzed in this paper differ greatly with respect to the results obtained by Baig et al. probably due to structure with a larger pore size distribution (within the range 130–190 μm, see Table 1). It should be pointed out that Baig et al. tested oils of different chemical composition and viscosity. At T = 40 °C, the kinematic viscosity of the three fluids is different, being 1.3–2.4 mm^2^/s for petroleum diesel, ≈0.56 mm^2^/s for toluene, and ≈0.42 mm^2^/s for hexane [49]. The different viscosity affects the absorption capacity of the samples. Furthermore, the measurement conditions of the present paper respected the ASTM F729-99 standard, while no indication is reported in Baig et al. All the considerations above discussed highlight as a comparation between our and Baig’s et al. work should be not strictly correct.

A comparison between the absorption data presented in this work and those already published in the literature has not been carried out. This is because it can be misleading due to the great variability of the boundary conditions used for its determination. These variables not only refer to the enormous diversity of the adsorbent materials tested, but mainly refer to the different measurement conditions and the different times in which the adsorption and squeezing test were performed. In fact, the tests are often carried out without a precise reference to the ASTM standards and with different non-polar liquids.

### 3.4. Fatigue Tests

Fatigue tests were carried out on the polyurethane foams, simulating continuous wringing cycles which are necessary to remove the absorbed oil, and, consequently, to highlight their mechanical performance.

**Figure 5:**
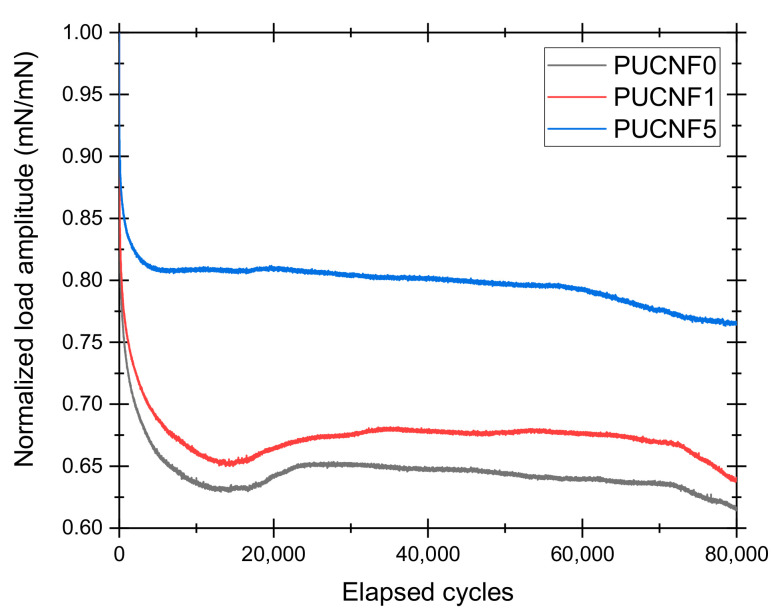
Normalized load amplitude of PCUNF0, PCUNF1, and PCUNF5 as a function of the elapsed cycles.

Figure 5 shows the effect of the elapsed cycles on the normalized load amplitude, i.e., the ratio between the load amplitudes.

PUCNF0 and PUCNF1 exhibited a similar behavior with a typical normalized load amplitude trend that can be divided into three branches: A first rapid descent, a second sufficiently constant, and a third in which the progressive reduction of mechanical performance occurs. Instead, the behavior of PUCNF5 is quite different from that of the other specimens. In fact, the presence of CNF facilitates the achievement of higher loads at the same displacement (−5 mm) and, therefore, an increase of Young’s modulus. The trend described above regarding the fatigue behavior of polyurethane foams agrees with some results reported in literature [43,44].

The area enclosed in the hysteresis cycle, represents the energy per unit of volume absorbed by the sample during the load application. In fact, it is the work produced by the action of forces and, for this reason, it appears as an interesting index to evaluate the reduction of the mechanical performance of the analyzed material.

**Figure 6:**
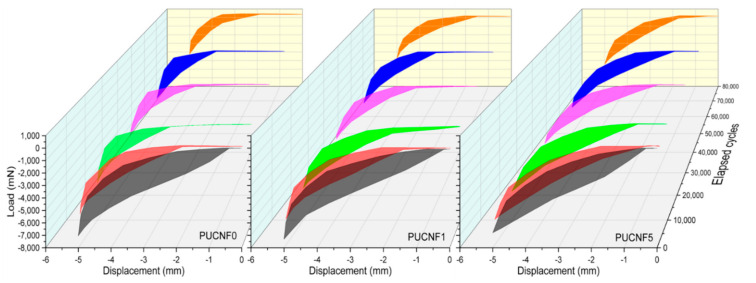
Development of the hysteresis behavior of the PUCNF0, PUCNF1, and PUCNF5.

Figure 6 illustrates the development of the hysteretic behavior of PCUNF0, PCUNF1, and PCUNF5.

**Figure 7:**
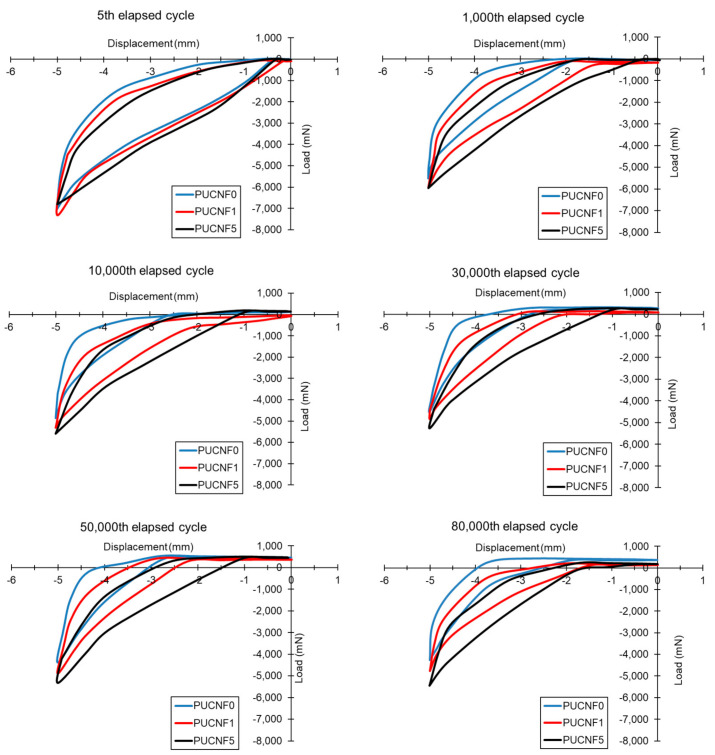
Comparison of the hysteresis behavior of PCUNF0, PCUNF1, and PCUNF5.

The increase of the elapsed cycles involves a reduction of the area contained by the hysteresis curve, i.e., of the elasticity, and a consequent increase in the plasticization of the material. This emphasizes the degradation of the samples. Figure 7 compares the hysteresis behavior of PCUNF0, PCUNF1, and PCUNF5 at the specific elapsed cycles.

At the first elapsed cycles, the three samples display a similar trend, however, after only 1000 cycles some differences can be identified. At 10,000 cycles, the hysteresis curve tends to shift towards lower compression loads and maintains a larger area as the amount of CNF present in the sponge increases. This attitude is best appreciated for many cycles and is maximized at 80,000 cycles.

The fatigue analyses showed that a greater filler content (5 wt%) increases the stiffness of the sponges and therefore delays the collapse of the foam cells compared to a lower CNF amount (1 wt%). Table 3 reports the percentage reduction of the maximum compression load for the three different samples as the number of elapsed cycles increases. Consistently with that previously reported, the addition of filler ensures a minor decrease in the maximum compression load. In particular, the specimen tends to exhibit an asymptotic trend for the percentage reduction after exceeding 10,000 cycles.

Although the literature provides several results for fatigue tests on polyurethane foams, according to the authors knowledge no work investigated the effect of a high number of cycles in the field of oil recovery. Furthermore, in the generic field of the polyurethane foams, the comparison of fatigue properties is not immediate since a univocal standard is not adopted [44,50,51,52].

**Figure 8:**
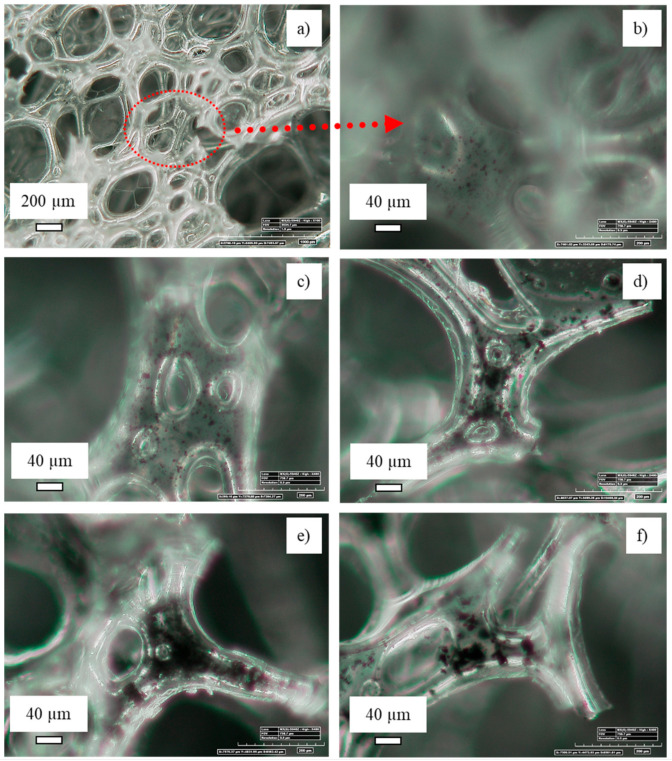
OM images of PUCNF0.5 at 100× (**a**) and its magnification at 400× (**b**); magnifications at 400× of PUCNF1 (**c**), PUCNF3 (**d**), PUCNF5 (**e**), and PUCNF15 (**f**).

The OM images of Figure 8 show the 400× enlargements of the various polyurethane sponge samples with various CNF content. The optical micrographs show that if the filler concentrations are small (0.5 and 1.0 wt%) its dispersion is homogeneous. By increasing the percentage content of the filler (≥3 wt%) there is an evident agglomeration effect of the nanoparticles which tend to form well-defined clusters, especially at a load of 15 wt%. These images, therefore, show that the filler can only be dispersed when it is added in small quantities.

Therefore, this result opens a space for reflection on the optimal quantity of carbonaceous filler because if on the one hand a high load creates coalescence and, therefore, uneven distribution with less selective absorbing power, on the other hand it stiffens the material making it more resistant to fatigue.

Hence, this paper represents the initial phase of the study of these materials, highlighting the need to improve the filler-matrix interaction, to act on the chemical composition, to optimize the quantity and distribution of the filler to reach a compromise between the selective absorption and the durability of the sponges.

## 4. Conclusions

In this work we have studied nanocomposite foams, polyurethane-based, prepared with a cheap carbonaceous filler (CNF) and with a facile synthetic route. Samples were characterized by physical and mechanical tests (contact angle measurement, absorption test according to ASTM F726-99 standard, optical microscopy observation, and compression fatigue tests according to ISO 24999 standard). Results indicated an increase in the hydrophobic behavior (θ = 111–114°) and a good oleophilic character of the composite sponges, besides an improved selective absorption of the foam toward oils in mixed water/oil media (+22.85%). The most performant filler amount was found around 1 wt% because of the homogeneous distribution inside the polymeric foam. Finally, the fatigue test results showed that the elastic behavior of the foam grows with the increasing carbon filler amount, thus achieving a relevant improvement in the long-term mechanical properties of the foams. In fact, after 80,000 fatigue cycles the load is reduced to 60% in the pure sponge while it is reduced to 80% in the sponge with 5 wt% of CNF. The presence of CNF leads to a stiffening of the sponge structure and to an increase of the energy per unit of volume, that is absorbed during the load application. This allows a greater resistance to fatigue, a growth of the elasticity, but certainly also a major required pressure to squeeze the sponge to extract the captured oil. Further studies will be dedicated to the optimization of the sponge composition, to obtain a nanocomposite with a high filler load that is in homogeneously dispersed and distributed in the polymer matrix. A possible solution could be a further size reduction of the CNFs by a ball milling, treating the particles for longer time.

In conclusion, the pros and cons of the developed materials can be summarized as follows:

- Materials obtainable with cheap raw materials and by a facile synthetic route;
- increase of hydrophobic behavior of PU with the addition of carbon nanofiber up to 1 wt%; and
- mismatch between the optimal filler load for oleophilic character and fatigue resistance.

## Figures and Tables

**Table 1:** Maximum, minimum, and average pore diameter of all the PUCNF foams.

	Pore Diameter (μm)		
Samples	Max	Min	Average
PUCNF0	213	38	115
PUCNF0.5	300	55	130
PUCNF1	350	40	140
PUCNF3	380	45	150
PUCNF5	400	45	190
PUCNF15	200	35	90

**Table 2:** Absorption capacity ratio in oil and water (Os, Ws) in pure (*) and mixed (**) systems.

	Absorption Capacity Ratio [g/g]	
Samples	Os/Ws *	Os/Ws **
PUCNF0	2.56	1.75
PUCNF0.5	2.29	1.94
PUCNF1	5.25	2.15
PUCNF3	4.3	1.95
PUCNF5	3.17	1.88

**Table 3:** Percentage reduction of the maximum compression load as a function of the elapsed cycles.

Sample	Elapsed Cycles					
	5th	1000th	10,000th	30,000th	50,000th	80,000th
PUCNF0	100%	78%	69%	64%	62%	60%
PUCNF1	100%	81%	72%	66%	67%	65%
PUCNF5	100%	87%	82%	77%	78%	80%

## Data Availability

Data are contained within this article.
